# A Test to Distinguish Monotone Homogeneity from Monotone Multifactor Models

**DOI:** 10.1007/s11336-023-09905-w

**Published:** 2023-03-18

**Authors:** Jules L. Ellis, Klaas Sijtsma

**Affiliations:** 1 Behavioural Science Institute, Radboud University Nijmegen, P.O.B. 9104, 6500 HE Nijmegen, The Netherlands; 2 Tilburg University, Tilburg, The Netherlands

**Keywords:** unidimensional measurement, multidimensional measurement, monotone latent variable model, monotone homogeneity model, conditional association

## Abstract

The goodness-of-fit of the *unidimensional* monotone latent variable model can be assessed using the empirical conditions of nonnegative correlations (Mokken in A theory and procedure of scale-analysis, Mouton, The Hague, 1971), manifest monotonicity (Junker in Ann Stat 21:1359–1378, 1993), multivariate total positivity of order 2 (Bartolucci and Forcina in Ann Stat 28:1206–1218, 2000), and nonnegative partial correlations (Ellis in Psychometrika 79:303–316, 2014). We show that *multidimensional* monotone factor models with independent factors also imply these empirical conditions; therefore, the conditions are insensitive to multidimensionality. Conditional association (Rosenbaum in Psychometrika 49(3):425–435, 1984) can detect multidimensionality, but tests of it (De Gooijer and Yuan in Comput Stat Data Anal 55:34–44, 2011) are usually not feasible for realistic numbers of items. The only existing feasible test procedures that can reveal multidimensionality are Rosenbaum’s (Psychometrika 49(3):425–435, 1984) Case 2 and Case 5, which test the covariance of two items or two subtests conditionally on the *unweighted* sum of the other items. We improve this procedure by conditioning on a *weighted* sum of the other items. The weights are estimated in a training sample from a linear regression analysis. Simulations show that the Type I error rate is under control and that, for large samples, the power is higher if one dimension is more important than the other or if there is a third dimension. In small samples and with two equally important dimensions, using the unweighted sum yields greater power.

**Supplementary Information:**

The online version contains supplementary material available at 10.1007/s11336-023-09905-w.

For binary test data satisfying a monotone item response theory (IRT) model, we develop a statistical test procedure that can detect multidimensionality as opposed to unidimensionality. Investigating the dimensionality of a psychological test is an important step in test development and validation. Establishing unidimensionality can contribute to construct validity of the test because this renders the interpretation of test performance easier, comparable with measurement in other science areas. Multidimensional item sets are edited by removing or replacing items deviating from the target attribute or splitting the item set in subsets representing better interpretable test performance. A case in point is the development of Spearman’s (1904) theory of general intelligence into the current multidimensional Cattell–Horn–Carroll (CHC) theory (Wasserman, 2019), based on psychometric analyses of numerous datasets.

Dimensionality analysis of an item set is usually done using factor analysis or IRT analysis (e.g., Sijtsma & Van der Ark, 2021). These approaches include parametric assumptions such as linearity and normality in factor analysis and logistic, normal-ogive, or step functions in IRT. These assumptions usually have little prior plausibility, which led several authors (Holland, 1981; Mokken, 1971; Rosenbaum, 1984; Stout, 1987) to study measurement using weaker assumptions, for example, replacing logistic and normal-ogive item response functions (IRFs) with monotone IRFs only subjected to order restrictions without choosing a parametric function.

The absence of restrictive parametric functions rendered the development of goodness-of-fit tests complex but was replaced with a focus on testable conditions that are the hallmark of an underlying quantitative variable. An example of a testable condition is that the inter-item correlations must be nonnegative (Mokken, 1971). The search for testable properties was also inspired by axiomatic measurement theory (Krantz et al., 1971) and probabilistic developments in it, such as the relation between simple scalability and strong stochastic transitivity in choice data (Tversky & Russo, 1969). This article also follows this approach.

We review two classes of monotone nonparametric IRT models and their testable conditions. Critical to this article, we argue that most conditions for which practical test procedures are available cannot distinguish multidimensional from unidimensional monotone IRT models. We target a specific set of covariance inequalities and demonstrate that we can use them to detect multidimensionality in cases that would previously remain undetected. We develop a practical test procedure and explore the Type I error rate and power using simulated data.

## Models and Testable Conditions

### Monotone Homogeneity and Monotone Factor Models

We discuss the definitions of three nonparametric IRT models that will be used throughout the article. The first model is monotone homogeneity (MH), which contends that the expected value of each observed binary item score variable increases with a single underlying variable, called the common factor, the latent variable, or the latent dimension. The second model, the monotone factor model (MFM), contends basically the same as the MH model with one or more independent factors to which the items are related in a simple structure. The third model is the higher-order monotone one-factor (HOMOF) model, which is like the MFM, but allows the factors to be correlated with the restriction that they depend on a single higher-order factor. The three models share the assumption of conditional (or local) independence or independent errors, which are similar assumptions. Thus, MH describes a general form of unidimensionality, MFM describes a general form of multidimensionality, and HOMOF is somewhere in between. Applied to intelligence, MH formally resembles Spearman’s theory of a single general intelligence factor, MFM is like Thurstone’s initial theory of multiple independent primary mental abilities, and HOMOF parallels the hierarchical factors of CHC, integrating the other two theories.

We assume the item scores are binary manifest variables, denoted ${{\textbf {X}}}=\left( X_{1}, \ldots ,X_{J} \right)$. Variable $X_{i}$ represents the scores (1 $=$ positive, 0 $=$ negative) subjects obtained on the *i*-th item. Factors, latent variables, or dimensions are denoted ${\varvec{\Theta }=}\left( {\Theta }_{1}, \ldots ,{\Theta }_{D} \right)$. The rest of this section discusses the formal definitions of the three models, which we need to prove the theorems.

We adopt the following assumptions (Holland & Rosenbaum, 1986; Mokken, 1971; Rosenbaum, 1984):

1. (conditional independence): ${{\textbf {X}}}$ is conditionally independent given ${\varvec{\Theta }}$.
2. (monotonicity): $P(X_{i}=1\vert \, \varvec{\Theta } )$ is an increasing function of ${\varvec{\Theta }}$ for all $i=1,\ldots ,J$.
3. (unidimensionality): $$D=1$$.

We use the term ‘increasing’ synonymous with ‘monotone nondecreasing’. For readers who do not have the information ready, Appendix A provides formulations of the assumptions having greater precision. Following Holland and Rosenbaum (1986), we will say that $({{\textbf {X}}}{,\, }{\varvec{\Theta }})$ is a monotone latent variable (MLV) model if MH1 and MH2 hold. Following Mokken (1971) Mokken and Lewis (1982) and Ellis and Junker (1997), we will say that ${{\textbf {X}}}$ satisfies a *unidimensional MLV model *or *MH model* if there exists a variable ${\Theta }$ such MH1, MH2, and MH3 hold.

Ellis (2015) studied a narrower class of monotone models. Slightly rephrasing Ellis, we will say that ${{\textbf {X}}}$satisfies an MFM if

$$\begin{aligned} {{\textbf {X}}}=\phi \left( \psi {\varvec{(\lambda \Theta }}\mathbf {)}+{\varvec{\upvarepsilon }} \right) , \end{aligned}$$

where $\phi$ and $\psi$ are component-wise increasing functions, ${\varvec{\Theta }}$ is a multivariate random vector with independent components, ${\varvec{\upvarepsilon }}$ is a multivariate random vector with components that are independent of each other and of ${\varvec{\Theta }}$, the $\upvarepsilon _{i}$s have log-concave densities (Appendix A), and ${\varvec{\lambda }}$ is a nonnegative real matrix with simple structure (i.e., every manifest variable loads positive on one factor and zero on the other factors). As an example of an MFM for binary manifest variables, consider a case where the $\upvarepsilon _{i}$s have standard normal distributions, $\psi$ is the identity function, and the $\phi _{i}$s are step functions with $\phi _{i}\left( x \right) =0$ if $x\le \beta _{i}$ and $\phi _{i}\left( x \right) =1$ if $x>\beta _{i}$ for some real number $\beta _{i}$. Then, $P\left( {X_{i}=1} \vert {\varvec{\Theta }}\right) ={\Phi }\left( \mathop {\sum }\nolimits _{d=1}^D {\lambda _{id}{\Theta }_{d}} -\beta _{i} \right)$, where ${\Phi (.)}$ is the standard normal distribution function. Hence, every multidimensional normal ogive IRT model with independent factors and nonnegative loadings with a simple structure is an MFM (also, see Takane & De Leeuw, 1987).

Ellis (2015) also studied a more general class of models, where the components of ${\varvec{\Theta }}$ need not be independent but may be the result of a higher-order MFM factor with log-concave disturbances at each level. We call this class of models, with possibly many levels and one factor at the highest level, a HOMOF model. In this class of models, the factor loadings at the lowest level (i.e., ${\varvec{\lambda }}$ in ${{\textbf {X}}}=\phi \left( \psi {\varvec{(\lambda \Theta }}\mathbf {)}+{\varvec{\upvarepsilon }} \right) )$ do not necessarily have simple structure.

### Testable Conditions of the Models

In this section, we review statistical inequalities that have been used to test whether MH holds for a given set of manifest variables. These inequalities can be expressed as covariances that are nonnegative. The general result implied by MH is conditional association (CA; Rosenbaum, 1984). Below, we discuss that CA is the hallmark of MH. Coincidentally, CA fares well with Spearman’s (Spearman, 1904) idea that intelligence tests have positive correlations and together measure a single general intelligence factor, and Guttman’s ‘first law of intelligence’, stating that any two intelligence items have a nonnegative correlation in any population that is “not artificially selected” (Guttman & Levy, 1991), thus suggesting the items should have nonnegative correlations in any subgroup defined by the other items. CA is hard to test fully because it involves many restrictions even for small item sets (De Gooijer & Yuan, 2011; Ligtvoet, 2022; Yuan & Clarke, 2001). Therefore, we will also discuss conditions that are easier to test, such as the condition that the expected item score increases with the rest score, called *marginal monotonicity *(MM) (Junker, 1993). These conditions can be viewed as incomplete tests of CA (Ligtvoet, 2022). We will now continue this section with the formal definitions.

Following Rosenbaum (1984), we say that ${{\textbf {X}}}$ is CA if for every partition ${{\textbf {X}}}=({{\textbf {Y}}},{{\textbf {Z}}})$ and every function *h*, for all increasing functions $\phi$ and $\psi$,

$$\begin{aligned} \textrm{Cov}\left( \phi \left( {{\textbf {Y}}} \right) ,\psi ({{\textbf {Y}}})\vert h\mathrm {(}\textbf{Z} \right) )\ge 0. \end{aligned}$$

Rosenbaum (1984) provides examples. Rosembaum’s result that

$$\begin{aligned} \textrm{MH}\Rightarrow \textrm{CA} \end{aligned}$$

is key to this article, in which we develop a practically feasible test for CA. Holland and Rosenbaum (1986) generalized Rosenbaum’s (Rosenbaum, 1984) work to non-binary variables. Ellis and Junker (1997; also Junker & Ellis, 1997) furthermore suggested that CA is sufficient to detect multidimensionality in a finite number of items. They studied infinite item sequences and used the condition of vanishing conditional dependence (VCD), which means that certain conditional covariances vanish as $J\rightarrow \infty$. They showed that CA and VCD are necessary and sufficient for a unidimensional monotone latent variable model in which the latent variable can be estimated consistently. Since VCD is defined only in the limit as $J\rightarrow \infty$, one would expect that if any condition can detect multidimensionality in a finite item set, it will be CA.

In addition to the practical infeasibility of CA due to large numbers of restrictions one must test even for small item sets, a complete test of CA is also impossible because of the sparseness of the data, because most item-score patterns never occur in the most commonly available sample sizes of 100 to 10,000 subjects. Maybe for this reason, many authors have studied weaker restrictions than CA, possibly believing they still capture the gist of CA. Straat et al. (2016) acknowledged this limitation and proposed an incomplete strategy. Ligtvoet (2022) gives an excellent review of weaker conditions, which he describes as “incomplete tests of conditional association”.

An important condition discussed by Ligtvoet (2022) is *multivariate total positivity of order 2 *(MTP$$_{2})$$. The ordinary formulation of this condition is given in Appendix A, but for the present purpose it suffices to note that $${{\textbf {X}}}$$ being MTP$$_{2}$$ implies that

$$\begin{aligned} \textrm{Cov}\left( \phi \left( {{\textbf {Y}}} \right) ,\psi ({{\textbf {Y}}})\vert {{\textbf {Z}}} \right) \ge 0. \end{aligned}$$

(Note the omission of *h*() here). Therefore, ${{\textbf {X}}}$ being MTP$_{2}$ means that $\textrm{Cov}\left( \phi \left( {{\textbf {Y}}} \right) ,\psi ({{\textbf {Y}}})\vert h\mathrm {(\textbf{Z}} \right) )\ge 0$ holds for some, *but not all*, functions *h* (Ellis, 2015, p. 264–265). For binary variables, the difference between CA and MTP$_{2}$ thus lies in the kind of events on which one may condition: MTP$_{2}$ involves conditioning on the finest partition of subgroups that can be made with ${{\textbf {Z}}}$, whereas CA also involves conditioning on combinations of such groups. Holland and Rosenbaum (1986) show that

$$\begin{aligned} \text {CA}\Rightarrow \text {MTP}_{2}. \end{aligned}$$

Therefore, a test of $\text {MTP}_{2}$ can be viewed as an incomplete test of CA (Ligtvoet, 2022).

For tests consisting of realistic numbers of items, however, MTP$_{2}$ still involves a large number of restrictions (Bartolucci & Forcina, 2005, p. 35; Ligtvoet, 2022). Therefore, one may want to reduce the number of restrictions to be tested even further by considering properties derived from MTP$_{2}$, which include nonnegative partial correlations (NPC; Ellis, 2015; Brusco et al., 2015) and nonnegative covariances (NNC) (Mokken, 1971).

The important testable property of Manifest Monotonicity (MM) (Junker, 1993; Junker & Sijtsma, 2000) means that each item regression on the sum of the other items (known as the rest score) is increasing (Appendix A). Junker (1993, p. 1372), Junker and Sijtsma (2000) showed that

$$\begin{aligned} \textrm{MH}\Rightarrow \textrm{MM}. \end{aligned}$$

Ligtvoet (2022) showed that $\textrm{CA}\Rightarrow \textrm{MM}$, so MM may be viewed as another incomplete test of CA. MM is an important property because it is conceptually like the idea of a monotone IRF, and for the reader who is unfamiliar with these concepts it may be hard to see how one can have MM without MH. Ellis (2014) gave an example where MM holds while NPC fails, and therefore, MH must fail too.

### Limitations of Partial Tests of Conditional Association

Ligtvoet (2022) concluded that existing incomplete tests of CA perform poorly detecting violations of CA. In the data structures he generated, CA was often violated while MTP$_{2}$ and MM held. Thus, a researcher who tests MTP$_{2}$ and/or MM instead of CA misses the violation of CA. We agree but notice that MTP$_{2}$ and MM are not sensitive to violations of dimensionality, necessitating the discussion of the problem from a more theoretical perspective. This discussion is partially inspired by Van den Wollenberg’s (Van den Wollenberg, 1982) proof that existing test statistics for the Rasch model were insensitive to violations of unidimensionality.

Ellis (2015; also proposition A1 of Appendix A) showed that for MTP$_{2}$ the problem is that,

$$\begin{aligned} \text {MFM}\Rightarrow \text {MTP}_{2}. \end{aligned}$$

Consequently, any test of MH based on MTP$_{2}$ (Bartolucci & Forcina, 2000, 2005) logically cannot distinguish MH from MFM. That is, if the test suggests that MTP$_{2}$ holds, it is also possible that an MFM that violates MH generated the data. The same conclusion holds for any property derived from MTP$_{2}$, which includes NPC (Ellis, 2015; Brusco et al., 2015), and NNC (Mokken, 1971). Thus, testing MTP$_{2}$, NPC, and NNC cannot distinguish unidimensional and multidimensional monotone factor models.

MM is implied by CA but not by MTP$_{2}$, and therefore, we need to discuss it separately. For MM, the problem is that

$$\begin{aligned} \textrm{MFM}\Rightarrow \textrm{MM}. \end{aligned}$$

This follows from Theorem 1 in Appendix B. Consequently, no test of MM (Douglas & Cohen, 2001; Junker & Sijtsma, 2000; Molenaar & Sijtsma, 2000; Tijmstra et al., 2013; Tijmstra & Bolsinova, 2019) can distinguish MH from MFM. That is, if the test suggests that MM holds, it is also possible that an MFM that violates MH generated the data. Thus, testing MM cannot distinguish unidimensional from multidimensional monotone factor models.

To summarize, based on data it is impossible to distinguish between MH and an MFM if one tests CA only partially with MTP$_{2}$, NNC, NPC, and MM. For example, assume that $J=10$, the first five items satisfying the Rasch model with latent variable ${\Theta }_{1}$, and the last five satisfying the Rasch model with latent variable ${\Theta }_{2}$, and ${\Theta }_{1}$ and ${\Theta }_{2}$ are independent. This is an MFM, thus satisfying MM and MTP$_{2}$, which implies NNC and NPC. Hence, it would be impossible to reject MH if only these conditions are tested. This puts the MH model at a serious disadvantage in comparison with parametric IRT models such as the two-parameter logistic model, where one can easily identify this situation via the M$_{2}$-test (Maydeu-Olivares & Joe, 2006).

The argument given includes the somewhat artificial case of independent factors, which implies that some items have correlation zero. One might argue that such cases would be excluded with Mokken’s (1971; Mokken & Lewis, 1982) criterion $H>.30$. However, Ellis (2015) also showed that

$$\begin{aligned} \textrm{HOMOF}\Rightarrow {\textrm{MTP}_{2}}. \end{aligned}$$

Although HOMOF models involve a single higher-order factor, there will generally be multiple first-order factors that are positively correlated with any degree. Consequently, it is possible that such items also satisfy $H>.30$.

We conclude that to distinguish unidimensional MFMs from multidimensional MFMs, the testable conditions MTP$_{2}$, NPC, NNC, and MM are logically insufficient. Therefore, we will target specific aspects of CA, which are covariance inequalities that will likely discriminate between MH and multidimensional MFMs. The next section discusses candidate covariance inequalities.

## Conditioning on Added Regression Predictions (CARP) Inequalities

### CARP Inequalities, Definition

Assume that all response probabilities of $${{\textbf {X}}}$$ are known; sample statistics will be discussed later. For a given pair ($$X_{i},X_{j})$$, denote by $${{\textbf {X}}}_{-ij}$$ the variables of $${{\textbf {X}}}$$ except $$X_{i}$$ and $$X_{j}$$. Our proposal is a generalization of Rosenbaum’s (1984, p. 427) “Case 2” method to test the covariance of each item pair conditionally on the rest score of the pair. For these covariances, CA implies that

$$\begin{aligned} \textrm{Cov}(X_{i},X_{j}\vert \mathop {\sum }\nolimits _{k\ne i,j}^J X_{k} )\ge 0. \end{aligned}$$

We call this the *conditioning on rest scores *(CRS)* inequality*, and we call a significance test for the CRS inequality a *CRS test*. A limitation of the CRS inequality for testing the dimensionality of a set of items is that the rest score used for conditioning is not adapted to the possible multidimensional structure if the item set does not satisfy MH. To obtain greater adaptation, we propose to use *weighted* rest scores. We use two linear regression analyses, where $X_{i}$ and $X_{j}$ serve as dependent variables and the other items are independent variables. Write $X_{0}:=1$, and denote the regression coefficient of $X_{k}$ in the prediction of $X_{i}$ from ${{\textbf {X}}}_{-ij}$ as $a_{k.ij}$, and denote the resulting predicted scores as $\hat{X}_{ij}$; that is,

$$\begin{aligned} \hat{X}_{ij}=\mathop {\sum }\nolimits _{k=0}^J {a_{k.ij}X_{k}}, \end{aligned}$$

where the $a_{k.ij}$ are such that they minimize $\mathbb {E}\left( \left( X_{i}-\hat{X}_{ij} \right) ^{2} \right)$, and with $a_{k.ij}=0$ if $k=i$ or $k=j$. Similarly, $\hat{X}_{ji}=\mathop {\sum }\nolimits _{k=0}^J {a_{k.ji}X_{k}}$ where the $a_{k.ji}$ are such that they minimize $\mathbb {E}\left( \left( X_{j}-\hat{X}_{ji} \right) ^{2} \right)$, and with $a_{k.ji}=0$ if $k=i$ or $k=j$. In other words, $\hat{X}_{ij}$ is the prediction of $X_{i}$ if $X_{j}$ is excluded as predictor, and $\hat{X}_{ji}$ is the prediction of $X_{j}$ if $X_{i}$ is excluded as predictor. As the basis for conditioning, we propose the variable

$$\begin{aligned} \hat{X}_{ij}+\hat{X}_{ji}=\mathop {\sum }\nolimits _{k=0}^J {(a_{k.ij}+a_{k.ji})X_{k}}. \end{aligned}$$

This is the sum of the predicted scores of $X_{i}$ and $X_{j}$, where both $X_{i}$ and $X_{j}$ are excluded from the predictors. It may be noted that we are not assuming linearity or normality here; we are just using the least squares solution as a heuristic tool, without claiming that this produces a good model.

The variable $\hat{X}_{ij}+\hat{X}_{ji}$ can attain many different values, producing small conditioning groups. Therefore, we will use deciles or other quantiles of this function. This is like Rosenbaum’s (1984, p. 428) “Case 5”, which tests the covariance of two subtests conditionally on deciles of the rest score. If $Q_{m}$ is an operator that divides any variable into *m* groups of approximately equal size, that is, $P[Q_{m}\left( X \right) =k]\approx m^{-1}$, then we obtain

$$\begin{aligned} \mathrm {Cov[}X_{i},X_{j}\vert Q_{m}\left( \hat{X}_{ij}+\hat{X}_{ji} \right) ]\ge 0. \end{aligned}$$

We refer to covariance inequalities of this form (with or without grouping by $$Q_{m})$$ as *conditioning on added regression predictions* (CARP) *inequalities*. Similarly, we refer to the involved conditional covariances as *CARP covariances*, and to the corresponding correlations as *CARP correlations*. We call the property that $${{\textbf {X}}}$$ satisfies all CARP inequalities, simply *CARP*.

CARP is the special case of CA with (if we use the notation used in the definition of CA) ${{\textbf {Y}}}=(X_{i},X_{j})$, $\phi \left( {{\textbf {Y}}} \right) =X_{i},\, \psi \left( {{\textbf {Y}}} \right) =X_{j},$ ${{\textbf {Z}}}={{\textbf {X}}}_{-ij}$, and $h\left( {{\textbf {Z}}} \right) =\, \mathop {\sum }\nolimits _{k=0}^J {(a_{k.ij}+a_{k.ji})X_{k}}$. The latter weighted sum is a function of ${{\textbf {X}}}_{-ij}$ because $X_{i}$ has weight $a_{i.ij}+a_{i. ji}=0$ and $X_{j}$ has weight $a_{j. ij}+a_{j.ji}=0$. We assume in this section that all response probabilities of ${{\textbf {X}}}$ are known, and therefore, the regression coefficients $a_{k.ij}$ are parameters, not sample statistics.

Hence, MH implies CARP. Furthermore, MFM does not imply CARP, which is demonstrated in later simulations, and which can also be seen in theoretical computations of some special cases. Therefore, testing CARP inequalities can reveal some violations of MH that testing MTP$_{2}$ or MM cannot reveal.

Let us now briefly explain why CARP inequalities may be useful in the assessment of multidimensionality. Suppose that $X_{i}$ and $X_{j}$ load on different independent latent variables, say, ${\Theta }_{1}$ and ${\Theta }_{2}$, and that the other items load on either ${\Theta }_{1}$ or ${\Theta }_{2}$. After a suitable transformation of ${\Theta }_{1}$ and ${\Theta }_{2}$, we may say that $\hat{X}_{ij}$ estimates ${\Theta }_{1}$ and $\hat{X}_{ji}$ estimates ${\Theta }_{2}$ (set ${\Theta }_{1}:=\mathbb {E}\left( \hat{X}_{ij} \vert {\Theta }_{1}\right)$ and ${\Theta }_{2}:=\mathbb {E}\left( {{\, }\hat{X}_{ji}} \vert {\Theta }_{2}\right) )$, so conditioning on $\hat{X}_{ij}+\hat{X}_{ji}$ tends to create groups with ${\Theta }_{1}+{\Theta }_{2}$ approximately equal, which induces a negative correlation between ${\Theta }_{1}$ and ${\Theta }_{2}$ in these groups (in groups where ${\Theta }_{1}+{\Theta }_{2}$ is constant, ${\Theta }_{2}$ is a decreasing function of ${\Theta }_{1})$, which in turn tends to create a negative correlation between $X_{i}$ and $X_{j}$. Theorem 2 of the Appendix states more formally that in this situation, the mean conditional covariance given the unweighted rest scores will be negative or zero, and Theorem 3 of the Appendix states that this will also be true for the mean conditional covariance given the weighted rest score $\hat{X}_{ij}+\hat{X}_{ji}$ provided that $\mathbb {E}\left( {{\, }X_{i}} \vert \hat{X}_{ij}\right)$ and $\mathbb {E}\left( {{\, }X_{j}} \vert \hat{X}_{ji}\right)$ are both increasing (i.e., the items have MM with respect to the partial weighted sum score of their respective subtest). In the standard two-dimensional case (defined in section 5.1) with ten items, we computed these correlations using numerical integration, and the outcomes supported our expectation that such correlations tend to be negative or zero. The simulations to assess the power of the CARP tests, reported later in this study, also support this result.

When we developed the test, we initially created a slightly different method, which can produce smaller correlations than the CARP correlations (the computation of the next example can be found in the Supplementary Material). For example, take two uncorrelated standard normal dimensions each with five Rasch items, all having $\beta _{i}=0$. Then, using numerical integration, one can obtain a correlation of -.204 in the union of the two most extreme vigintile groups of $\hat{X}_{ij}-\hat{X}_{ji}$; that is, $\varphi \left( {X_{i},X_{j}} \vert {[Q_{20}\left( \hat{X}_{ij}-\hat{X}_{ji} \right) =1]\, \cup [Q_{20}\left( \hat{X}_{ij}-\hat{X}_{ji} \right) =20]}\right) =-.204$. This is not aCARP correlation, because we condition on $\hat{X}_{ij}-\hat{X}_{ji}$ instead of $\hat{X}_{ij}+\hat{X}_{ji}$. Although this correlation is smaller than the CARP correlations we obtained, a statistical test based on this conditional correlation of $-.204$ turns out to be less powerful because the 90% observations with $2\, \le \, Q_{20}\left( \hat{X}_{ij}-\hat{X}_{ji} \right) \le 19$ are discarded. Simulations showed that a CARP test has greater power in this case. The next section focusses on a test statistic that can be used to test CARP.

Our approach is almost the opposite of the DETECT and DIMTEST procedures for investigating an item set’s dimensionality (Stout et al., 1996; Zhang & Stout, 1999a, b). DETECT and DIMTEST look for large conditional covariances, averaged over item pairs, as a sign that unidimensionality is violated. Unlike CARP, these approaches do not use rigorously established inequalities for the conditional covariances, but rather assume that they are approximately equal to certain theoretical conditional covariances given ${\Theta }$.

## A Statistical Test of CARP for a Single Focal Pair

We develop a significance test that we can use to check whether the CARP inequality holds for a single pair ($$X_{i},X_{j})$$, called the *focal* pair. We discuss computation in six steps. The algorithm will become available in the R-package mokken (Van der Ark, 2007, 2012). Analyzing 100,000 samples with $$N=10000$$ and $$J=10$$ took less than 6 min in total.

*Step 1: Select a focal item pair*. We propose four strategies:

1. If the researcher suspects different items measure different attributes, pick one item representative of one attribute and another item representing another attribute. For example, some arithmetic items including item *i* may also measure a verbal attribute and others including item *j* a nonverbal attribute. Pick item *i* and item *j*.
2. If data are available from previous research, an explorative analysis may be done using factor analysis or a parametric multidimensional IRT model. If different dimensions appear, again pick two items each representing another attribute. For example, in a factor solution, not necessarily from a well-fitting model, items can be selected that load high on one factor and close to zero on another factor, thus providing a heuristic tool.
3. The CARP procedure involves splitting the sample into training and test samples. The training sample can be used to select the focal pair in the same way as in Strategy 2.
4. Let $(X_{i},X_{j})$ run over all possible pairs ($X_{1},X_{2}),\, \left( X_{1},X_{3} \right) \mathrm {,\, \ldots ,\, }\left( X_{2},X_{3} \right) ,\, \left( X_{2},X_{4} \right) , \ldots$ and apply the test to each pair. A later section discusses methods to combine multiple item pairs.

*Step 2: Select a training sample*. Split the total sample of *N* subjects randomly into a training sample of *L* subjects and a test sample of *M* subjects ($$N=L+M)$$. The proportion of subjects in the training sample is $$\ell =L/N$$. We use the training sample to estimate the regression coefficients and use these in the test sample to compute the test statistic. Based on simulation work reported later, for small samples ($$N\le 500)$$, we recommend $$\ell =.5$$, and for larger samples $$\ell =.2$$ or $$\ell =.3$$.

*Step 3: Estimate the regression coefficients*. Linear regression analysis on the training sample yields estimates of the coefficients $a_{k.ij}$ and $a_{k.ji}$, denoted $\hat{{{a}}}_{k.ij}$ and $\hat{{ {a}}}_{k.ji}$ (with $\hat{{ {a}}}_{i.ij}=\hat{{ {a}}}_{j.ij}=0)$.

*Step 4: Estimate quantiles of the predicted scores*. Using only the training sample, compute the estimated predicted scores

$$\begin{aligned} \hat{\hat{X}}_{ij}=\mathop {\sum }\nolimits _{k=0}^J {\hat{{{a}}}_{k.ij}X_{k}}, \end{aligned}$$

and similarly, for $$\hat{\hat{X}}_{ji}$$. Next, determine the empirical distribution function of $$\hat{\hat{X}}_{ij}+{\, }\hat{\hat{X}}_{ji}$$ in the training sample. The distribution is used to define *m* quantiles. Here, we propose using deciles ($$m=10)$$. Thus, the outcome of Step 4 is a list of real numbers $$\hat{{{q}}}_{1\, ij}<\, \hat{{{q}}}_{2\, ij}<\ldots <\, \hat{{ {q}}}_{9\, ij}$$ such that

$$\begin{aligned} P\left( \hat{{{q}}}_{(s-1)\, ij}<\hat{\hat{X}}_{ij}+{\, }\hat{\hat{X}}_{ji}\le {\, }\hat{{{q}}}_{s\, ij} \right) \approx 0.1 \end{aligned}$$

for $s=\, 1,\, \ldots ,\, 10$, where we write $\hat{{{q}}}_{0\, ij}=-\infty$ and $\hat{{ {q}}}_{10\, ij}=\infty$. The precise algorithm used in the simulations is provided in the Supplementary Material.

*Step 5: Compute the conditioning variable in the test sample*. Using the estimated regression coefficients $\hat{{{a}}}_{k.ij}$ and $\hat{{ {a}}}_{k.ji}$, and the estimated quantile separators $\hat{{{q}}}_{s\, ij}$ estimated in the training sample, we extend the computation of $\hat{\hat{X}}_{ij}+{\, }\hat{\hat{X}}_{ji}$ to the test sample. Next, we compute the conditioning variable $C_{ij}$ in the test data by

$$\begin{aligned} \hat{{ {q}}}_{(s-1)\, ij}<\hat{\hat{X}}_{ij}+{\, }\hat{\hat{X}}_{ji}\le {\, }\hat{{ {q}}}_{s\, ij}\Longleftrightarrow C_{ij}=s \end{aligned}$$

*Step 6: Compute the one-sided Mantel–Haenszel Z*. Using the test sample, test the null hypothesis that $Cov({{X}_{i}},{{X}_{j}}|C_{ij}=s)\ge 0$ for $s=1,\, 2,\, \ldots ,\, m$ by means of a one-sided version of the Mantel–Haenszel statistic. We will use the test proposed by Rosenbaum (1984, p. 429; see Kuritz et al., 1988, for a discussion of different versions of the Mantel–Haenszel method). The following description is copied almost verbatim from Rosenbaum: Denote the number of subjects in the test sample having $X_{i}=a$, $X_{j}=b$, and $C_{ij}=s$ as $n_{abs}$ for $a,b=0,\, 1;s=1,\, 2,\, \ldots ,\, m$ and denote the marginal totals as $n_{+bs}=n_{0bs}+n_{1bs}$, $n_{a+s}=n_{a0s}+n_{a1s}$, $n_{ab+}=\sum \nolimits _s {{\, }n_{abs}}$, etc. Compute

$$\begin{aligned} e_{+}= & {} \mathop {\sum }\nolimits _{s=1}^m \frac{n_{1+s}n_{+1s}}{n_{++s}},\\ v_{+}= & {} \mathop {\sum }\nolimits _{s=1}^m \frac{n_{1+s}n_{0+s}n_{+1s}n_{+0s}}{n_{++s}^{2}(n_{++s}-1)}, \end{aligned}$$

and then the test statistic

$$\begin{aligned} Z_{ij}=\frac{n_{11+}-e_{+}+0.5}{\sqrt{v}_{+} }. \end{aligned}$$

The *p*-value is computed as $p_{ij}={\Phi }^{-1}(Z_{ij})$, where ${\Phi }^{-1}(.)$ is the inverse of the standard normal distribution function. See Rosenbaum (1984, p. 429) for more details and the rationale of $Z_{ij}$. The sample covariance of $X_{i}$ and $X_{j}$ in the layer with $C_{ij}=s$ is given by

$$\begin{aligned} \widehat{cov}({{X}_{i}},{{X}_{j}}|C_{ij}=s) =\frac{n_{11s}}{n_{++s}}-\frac{n_{1+s}}{n_{++s}}\frac{n_{+1s}}{n_{++s}} \end{aligned}$$

and therefore,

$$\begin{aligned} n_{11+}-e_{+}=\mathop {\sum }\nolimits _{s=1}^m {n_{++s}\widehat{cov}({{X}_{i}},{{X}_{j}}|C_{ij}=s)}. \end{aligned}$$

The numerator of $Z_{ij}$ is therefore a weighted sum of the conditional covariances of $X_{i}$ and $X_{j}$, given the grouped weighted rest scores, with a continuity correction. Rosenbaum noticed that the quantities $e_{+}$ and $v_{+}$ are the expectation and variance of $n_{11+}$ in the least favorable case of the null hypothesis, which is the case where $Cov({{X}_{i}},{{X}_{j}}|C_{ij}=s)=0$ for $s=1,\, 2,\, \ldots ,\, m$. If the null hypothesis is true, then $Cov({{X}_{i}},{{X}_{j}}|C_{ij}=s)\ge 0$ and $\mathbb {E}\left( n_{11+}-e_{+} \right) \ge 0$, so that $Z_{ij}$ has an asymptotic normal distribution with $\mathbb {E}(Z_{ij})\ge 0$.

The optimal number (*m*) of quantile groups in Step 4 is rather arbitrary in the sense that there is no definitive number. Rosenbaum (1984) suggested deciles ($$m=10)$$ in his “Case 5”, but also used the raw rest score, which has $$J-2$$ levels. We did simulations with both linear and logistic regression, with $$m=10$$, $$m=J-2$$, and $$m=\sqrt{N} $$. The differences in power between these versions were small, but linear regression with $$m=10$$ had slightly higher power than the other options. Therefore, we use linear regression with $$m=10$$ in all simulations below.

## Asymptotic Type 1 Error Rate

We provide a formal proof that the Type 1 error rate is under control as $N\rightarrow \infty$. Note that in Step 6 we suggested a one-sided version of a Mantel–Haenszel test, but that multiple versions of the Mantel–Haenszel test exist (Kuritz et al., 1988), and more versions can be developed in the future. We want a result that is valid for all these versions, and rather than delving into the details of each possible version, we will make the general assumption that in Step 6 one applies a test with the following property: If the test is applied to data of a $2\times 2\times K$ table to test the null hypothesis that the covariance is nonnegative in each of the *K* layers, then the asymptotic Type 1 error rate is under control in the sense that the *p*-values stochastically dominate a standard uniform random variable as the sample size grows to infinity. Now, the question is whether that remains true in our case, where the layers are partially based on the regression estimated from a training sample rather than on a fixed variable in the test sample.

### Proposition 1

If subjects are drawn randomly and independently and if the test sample grows infinitely large while the training sample remains fixed, then the asymptotic Type 1 error rate of the CARP test is under control.

### Proof

Denote the data of the *L* subjects of the training sample ${{\textbf {X}}}^{(1)}=({{\textbf {X}}}_{1}^{\left( 1 \right) },\, {{\textbf {X}}}_{2}^{\left( 1 \right) },\ldots ,{{\textbf {X}}}_{L}^{\left( 1 \right) })$ and the data of the *M* subjects of the test sample ${{\textbf {X}}}^{(2)}=({{\textbf {X}}}_{1}^{\left( 2 \right) },\, {{\textbf {X}}}_{2}^{\left( 2 \right) },\ldots ,{{\textbf {X}}}_{M}^{\left( 2 \right) })$. Subjects are drawn randomly and independently, therefore we consider the random vectors ${{\textbf {X}}}_{n}^{\left( t \right) }$ as independent and identically distributed (iid) copies of ${{\textbf {X}}}$. The *k*-th item score in ${{\textbf {X}}}_{n}^{\left( t \right) }$ is denoted $X_{nk}^{\left( t \right) }$. Define *g*(.) to be the function such that, for any vectors ${\varvec{\alpha }}{\in }\mathbb {R}^{2(J+1)}$ and ${\varvec{\beta }}\in \mathbb {R}^{m-1}$ with $\beta _{1}<\beta _{2}<\ldots <\beta _{m-1}$, if we write $\beta _{0}=-\infty$ and $\beta _{m}=\infty$, then

$$\begin{aligned} \beta _{s-1}<\, \mathop {\sum }\nolimits _{k=0}^J {(\alpha _{k}+\alpha _{k+J+1})X_{k}} \le {\, }\beta _{s}\Longleftrightarrow g\left( {{\textbf {X}}},{\varvec{\alpha }},{\varvec{\beta }} \right) =s, \end{aligned}$$

for $s=1,\, 2,\, \ldots ,\, m$. This definition parallels the definition of the conditioning variable $C_{ij}$, given in the description of Step 5. We denote the vectors of estimated regression coefficients and quantile separators $\hat{{\textbf {a}}}_{ij}\left( {{\textbf {X}}}^{(1)} \right)$ and $\hat{{\textbf {q}}}_{ij}\left( {{\textbf {X}}}^{(1)} \right)$, respectively, so that we can write the conditioning variable for the *n*-th subject in the test sample as

$$\begin{aligned} C_{ijn}=g\left( {{{\textbf {X}}}}_{n}^{(2)},{\, }\hat{{{\textbf {a}}}}_{ij}\left( {{{\textbf {X}}}}^{(1)} \right) \text {,}\hat{{{\textbf {q}}}}_{ij}\left( {{{\textbf {X}}}}^{(1)} \right) \right) . \end{aligned}$$

Now, consider the conditional covariance of the form $Cov({{X}_{i}},{{X}_{j}}|C_{ij}=s)$. For the *n*-th subject in the test sample, the corresponding covariance is $Cov\left( X_{ni}^{(2)},X_{nj}^{(2)}\vert C_{ijn}=s \right)$. Consider the latter covariance conditionally on ${{\textbf {X}}}^{(1)}$. Given ${{\textbf {X}}}^{(1)}$, ${{\textbf {X}}}_{n}^{(2)}$ is conditionally associated (because ${{\textbf {X}}}$ is conditionally associated and ${{\textbf {X}}}_{n}^{(2)}$ is a copy of ${{\textbf {X}}}$ that is independent of ${{\textbf {X}}}^{(1)})$. Furthermore, given ${{\textbf {X}}}^{(1)}$, $C_{ijn}$ depends only on ${{\textbf {X}}}_{n}^{(2)}$ with the two variables $X_{ni}^{\left( 2 \right) }$ and $X_{nj}^{\left( 2 \right) }$ excluded (since we required $\hat{{ {a}}}_{i. ij}=\hat{{{a}}}_{j. ij}=0)$, and therefore, $Cov\left( X_{ni}^{(2)},X_{nj}^{(2)}\vert C_{ijn}=s,{{\textbf {X}}}^{(1)} \right) {\, }{\, }$is implied to be nonnegative by conditional association of ${{\textbf {X}}}_{n}^{(2)}\vert {{\textbf {X}}}^{(1)}$. Nonnegativity holds for $n=1,\, 2,\, \ldots ,\, M$. It can be concluded that the data of the test sample can be considered as *M* independent draws from a population with $Cov({{X}_{i}},{{X}_{j}}|C_{ij},\, {{\textbf {X}}}^{(1)})\, \ge 0$. Therefore, the asymptotic distribution of $p_{ij}\vert {{\textbf {X}}}^{(1)}$ dominates the uniform (0, 1) distribution in the sense that for any $\alpha \in (0,1)$, $\mathop {\text{ lim } \text{ sup }}\limits _{M\rightarrow \infty }P(p_{ij}<\alpha |{{{\textbf {X}}}}^{(1)}) \le \alpha$. The decision rule “reject the null hypothesis if $p_{ij}<\alpha "$ will thus lead to asymptotic Type 1 error rate $\mathop {\text{ lim } \text{ sup }}\limits _{M\rightarrow \infty }{{P(p}_{ij}<\alpha )}=\mathop {\text{ lim } \text{ sup }}\limits _{M\rightarrow \infty }{\mathbb {E}({P(p}_{ij}<\alpha \vert {{{\textbf {X}}}}^{\left( 1 \right) }))}$, and with the reverse Fatou lemma we have that this is $\le \mathbb {E}\, \mathop {\hbox {lim sup}}\limits _{M\rightarrow \infty }{{P(p}_{ij}<\alpha \vert {\textbf {X}}^{\left( 1 \right) })}\le \alpha$ $\square$

Proposition 1 holds no matter how poor the estimates $\hat{{\textbf {a}}}{,}{\, }\hat{{\textbf {q}}}$ are or how much off-target the heuristic tool is. All that is needed to control the Type 1 error rate is that the subjects are drawn iid, that the estimates $\hat{{\textbf {a}}}{,}{\, }\hat{{\textbf {q}}}$ are based on the training sample, that the training sample is independent of the test sample, and that the weights of the focal variables are fixed to zero in $\hat{{\textbf {a}}}$.

Proposition 1 assumes that the size of the training sample remains fixed while the size of the test sample increases. If, however, *L* and *M* increase simultaneously, we presumably also need that $\hat{{\textbf {a}}}$ and $\hat{{\textbf {q}}}$ converge as $L\rightarrow \infty$, because $C_{ijn}$ depends on ${{\textbf {X}}}^{(1)}$ and therefore, on *L*. If $\hat{{\textbf {a}}}$ and $\hat{{\textbf {q}}}$ converge, then the $C_{ijn}$s stabilize, and we expect that the proof can be modified to establish that the Type 1 error rate is under control in this situation as well. However, we see no point in dwelling on cases with $L\rightarrow \infty$, because increasing the training sample has almost no benefits once the standard errors of $\hat{{\textbf {a}}}$ and $\hat{{\textbf {q}}}$ become very small. For all practical purposes we can therefore add to our procedure the prescription to cap *L* when the estimated standard errors of $\hat{{\textbf {a}}}$ and $\hat{{\textbf {q}}}$ are below a certain small threshold. This happens almost surely for large *L* if these estimates are obtained by linear regression and the empirical distribution function, as we discussed in the previous section. Then, the proof suffices to establish asymptotic Type 1 error rate control.

We explored whether the test can be modified such that the training sample and the test sample both include the whole sample, but simulations showed that this modification causes the Type I error rate to exceed the nominal significance level in some cases. Hence, we recommend cross-validation.

## Simulation Studies

### Method

#### General Set-Up

We used *J* items and a logistic model, $P\left( X_{i}=1\vert {\Theta }_{1},{\Theta }_{2},{\Theta }_{3} \right) =\left( 1+\exp \left( -\left( \alpha _{i1}{\Theta }_{1}+\alpha _{i2}{\Theta }_{2}+\alpha _{i3}{\Theta }_{3}+\beta _{i} \right) \right) \right) ^{-1}$, where ${\mathrm {(\Theta }}_{1},{\Theta }_{2},{\Theta }_{3})$ has a trivariate standard normal distribution with correlations 0. Denote the number of items that load on dimensions 1, 2, and 3 as $J_{1}$, $J_{2}$, and $J_{3}$, respectively, so that $J_{1}+J_{2}+J_{3}=J$. We distinguish the *standard two-dimensional case* as a special case with $\alpha _{id}=1$ if item *i* loads on dimension *d*, and $\alpha _{id}=0$ otherwise, $\beta _{i}=0$, and $J_{1}=J_{2}$ and $J_{3}=0$. We call this the ‘standard’, but it represents a failure if the goal was to create a unidimensional test that satisfies MH.

#### Optimum Size of Training Sample

Training samples in cross-validation often contain at least 70% of the observations of the whole sample. We did some simulations to find out whether we must maintain that percentage here. We used the standard two-dimensional case with $$J\in \{12,\, 24\}$$ and $$N\in \{500,\, 1000,\, 2000,\, 5000\}$$, and $$\ell \in \{.1,\,.2,\,.3,\,.4,\,.5,.6,.7,\,.8,.9\}$$, using 1000 samples per $$(J,N,\, \ell )$$ cell. For each combination of *J* and *N*, we fitted the power of the CARP method found in the simulation by a quadratic regression on $$\ell $$. From the estimated regression coefficients, we computed the value of $$\ell $$ for which the quadratic curve has its maximum. Table 1 shows that for small samples ($$N=500)$$, the estimated optimum was close to $$\ell =.5$$, and for large samples the optimum was rather $$\ell =.2$$ or $$\ell =.3$$.

**Table 1:** Estimated optimum values of training proportion $\ell$ for varying sample size *N* and test length *J*.

	*J*	
*N*	12	24
500	.45	.48
1000	.38	.43
2000	.31	.36
5000	.19	.31

## Results

### Type I Error Rate

For large samples, the Type I error rate is under control because of the asymptotic properties of the Mantel–Haenszel test. It suffices to study the error rate for small samples, and we focused first on $N=500$ with $\ell =.3$ for this purpose. In unidimensional cases, we chose $\alpha _{i2}=\alpha _{i3}=0$ for $i=1,\ldots ,J$. We focused on cases with $\alpha _{i1}=\alpha _{i2}=\alpha _{i3}=0$, which we refer to as zero-dimensional. (One can also describe this case as *J*-dimensional, but the number of common dimensions would still be 0.) These cases are interesting because all CARP covariances are zero, whereas they are positive in the unidimensional case with positive loadings. Consequently, the rejection rates were generally higher in zero-dimensional cases than in other unidimensional cases. Parameters that were not fixed to 0 were randomly drawn from the following distributions: $\beta _{i}\, \sim \, Uniform(-1.5,\, 1.5)$ and $\alpha _{i1}\, \sim \, Uniform(0.5,\, 2.5)$. We studied the effect of the number of items, *J*, varying between 10 and 50. For each *J*, we simulated 100 parameter sets *S*, each consisting of $(\alpha _{i1},\, \beta _{i})$, $i=1,\ldots ,J$. Next, for each of the 100 parameter sets we simulated 1000 samples of *N* subjects responding to the *J* items and applied the CARP test procedure to this sample with nominal significance level $\alpha =.05$. Thus, for each *J* we have 100 parameter sets, and for each parameter set, we obtained a rejection rate based on 1000 samples.

**Fig. 1:**
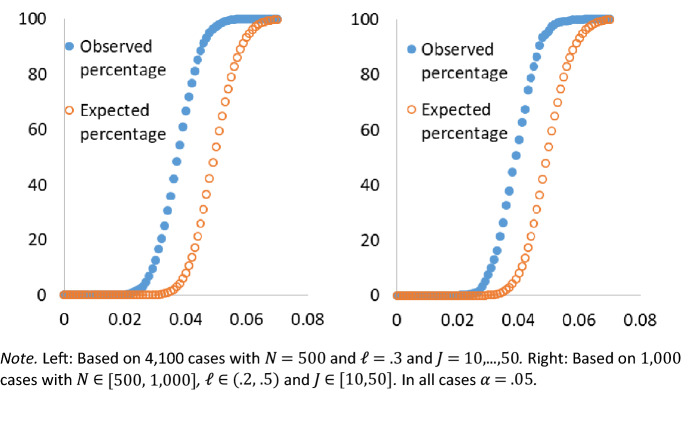
Cumulative Percentages of Type I Error Rates in Zero-Dimensional Cases.

Table 2 shows the quartiles of the rejection rates with $\ell =.3$ for some selected values of *J*. The maximum rejection rate over all 4100 zero-dimensional cases (41 values of *J*, each with 100 cases of 1000 samples) was .065, which is not significantly larger than .05 according to a binomial test with multiple testing correction (for a single case of 1000 samples, the *p*-value would be $1-pbinom\left( 65,1 000,.05 \right) =.0149$, but for the maximum of 100 cases the *p*-value is $1-{pbinom\left( 65,1 000,.05 \right) }^{4100}=1)$. The mean rejection rate was .038. Figure 1 shows the cumulative percentages of the rejection rates along with the expected cumulative percentages derived from a binomial distribution with success probability .05. The expected distribution clearly dominates the distribution of rejection rates. Therefore, we conclude that the Type I error rate of the CARP test is under control in these cases.

**Table 2:** Type I error rates in zero-dimensional cases.

	*J*				
Quartile	10	20	30	40	50
0 (minimum)	.018	.024	.019	.025	.022
1	.032	.034	.034	.032	.033
2	.038	.038	.038	.037	.038
3	.041	.040	.043	.041	.041
4 (maximum)	.052	.052	.055	.051	.051

Each column is based on 100 cases with 1000 samples each, with ${\ell =.3}$ and $N\mathrm {=500}$.

**Table 3:** Rejection Rates of CARP and CRS Tests in Two-Dimensional Cases with $N=$ 5000.

*J*	$J_{1}$	$J_{2}$	$J_{3}$	CARP	CRS
12	2	10	0	**.363**	.231
12	3	9	0	**.601**	.533
12	4	8	0	.727	**.797**
12	5	7	0	.792	**.929**
12	6	6	0	.791	**.949**
24	2	22	0	**.320**	.118
24	3	21	0	**.627**	.234
24	4	20	0	**.753**	.450
24	5	19	0	**.836**	.680
24	6	18	0	**.889**	.839
24	7	17	0	.892	**.913**
24	8	16	0	.937	**.970**
24	9	15	0	.929	**.989**
24	10	14	0	.950	**.995**
24	11	13	0	.945	**.995**
24	12	12	0	.954	**.998**

Values in bold are the largest power in the row

Each row is based on 1000 samples.

We also did simulations where $\mathrm {\ell },\, N$, and *J* were randomly drawn from a uniform distribution, with $\mathrm {\ell }\in (.2,\,.5)$, $N{\in [500,\, 1000]}$, and $J{\in [10,\, 50]}$. We simulated zero-dimensional parameter cases with 1000 samples each. The maximum rejection rate was .062, which is not significant according to a binomial test with multiple testing correction $(p=1-{pbinom\left( 62,1000,0.05 \right) }^{1000}=1)$. The mean rejection rate was .040. Figure 1 shows the cumulative frequencies of the rejection rates along with the expected cumulative frequencies derived from a binomial distribution with success probability .05. The binomial distribution clearly dominates the distribution of the rejection rates. Therefore, we conclude that the Type I error rate is under control. The simulations with random $\mathrm {\ell },\, N$, and *J* were also conducted for unidimensional cases with $\alpha _{i1}\, \sim \, Uniform(0.5,\, 2.5)$. These rejection rates were all well below .05.

Finally, we did some simulations with $N\gg 500$ and small *J*. Both zero-dimensional and unidimensional cases were simulated with $\ell \in (.2,\,.5)$, $N{\in \{{5000, 10000, 20000}\}}$, and $J{\in \{5,\, 10\}}$ with 10 parameter cases per cell and 1000 samples per parameter case. The rejection rates were again around .05 in the zero-dimensional cases, and close to 0 in the unidimensional cases.

### Power: Single Focal Pair, Effect of Dimensionality, and Item Distribution

We chose $\alpha _{id}=1$ if item *i* loads on dimension *d*, and $\alpha _{id}=0$ otherwise. We used $\beta _{i}=0$. Consider the standard two-dimensional case where $J_{1}=J_{2}$ and $J_{3}=0$. For large *N*, if linear regression is used, $\hat{X}_{ij}+\hat{X}_{ji}$ converges to a linear transform of the rest score $(\sum \nolimits _k {X_{k})} -X_{i}-X_{j}$. Therefore, the power of the CARP test will approach that of a CRS test. However, for finite *N*, the power of the CARP test will remain below that of the CRS test, because part of the sample is used for training and not for testing, and because $\hat{X}_{ij}+\hat{X}_{ji}$ is not exactly equal to the rest score yet. This was indeed what we found in simulations. Therefore, one may consider the standard two-dimensional case as the ideal case for Rosenbaum’s CRS test. Next, we will compare the power of the CARP test and the CRS test in various deviations from this standard case. The first kind of deviations is that $J_{1}\ne J_{2}$. The second kind of deviation is that $J_{3}>0$, introducing items that load on a third dimension that we assume uncorrelated with the other two dimensions. These simulations were conducted with all *J* between 9 and 39 that are multiples of 3, but we report results in detail only for $J=12$ and $J=24$.

Table 3 shows the CARP test’s power with $N=5000,$ and $\ell =.2$ for cases with $J=12$ or $J=24$, and $J_{3}=0$. The CARP test had significantly greater power than the CRS test in the seven cases with $J_{1}/J\, <.27\,$or $J_{1}/J>.73$ and in some of these cases, the power of the CARP test was considerably larger. In the other nine cases, the CRS test had more power, but the power of the CARP test was rather close to it. In general, the CARP test had greater power. The results for other values of *J* were similar: For $J_{1}/J\, <.27\,$or $J_{1}/J>.73$, the CARP test had significantly greater power than the CRS test. For $J_{1}/J\, \, \,$between .30 and .70, the CARP test had significantly smaller power than the CRS test. For values of $J_{1}/J$ between .27 and .30, or between .70 and .73, the difference in power between the CARP and CRS tests was usually not significant.

Table 4 shows the power with $N=5000,$ and $\ell =.2$ for cases with $J=12$ or $J=24$, $J_{1}=J_{2},\,$and $J_{3}>0$. The CARP test had greater power than the CRS test in the ten cases with $J_{1}=J_{2}\le J_{3}$, and in some of these cases the power of the CARP test was considerably greater. In the other four cases with $J_{1}=J_{2}>J_{3}$, the CRS test had greater power, but the power of the CARP test was still substantial. In general, the CARP test had greater power. Results for other values of *J* were similar. For small *N*, the CARP test lost power compared to the CRS test, because the training sample was excluded from the test. Thus, the results for smaller *N* were more favorable for the CRS test.

### Power: Single Focal Pair, Effect of Item Parameters and Sample Size

**Fig. 2:**
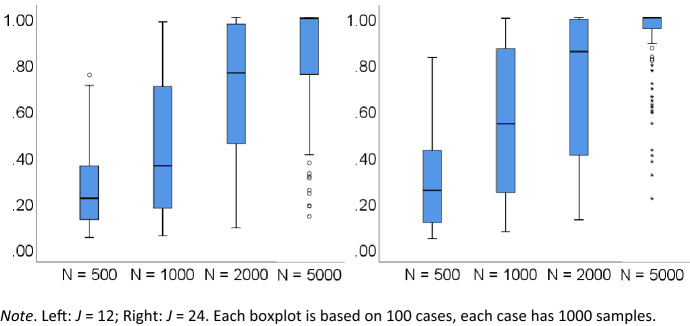
Rejection rates in two-dimensional cases with different item parameters.

We studied two-dimensional tests with $J_{1}=J_{2}$ and $J_{3}=0$, with $N=500,\, 1000,\, 2000,\, 5000$, $\ell =.3$, and $J=12$ or $J=24$. We chose $\alpha _{i2}=0$ for $i=1,\ldots ,J_{1}$ and $\alpha _{i1}=0$ for $i=J_{1}+1,\ldots ,J$. Parameters that were not fixed to 0 were randomly drawn from the following distributions: $\beta _{i}\, \sim \, Uniform(-1.5,\, 1.5)$ and $\alpha _{i1},\, \alpha _{i2}\, \sim \, Uniform(0.5,\, 2.5)$. For $J=12,\, 24$, we simulated 100 parameter sets *S*, each consisting of $(\alpha _{i1},\, \alpha _{i2},\, \beta _{i})$, $i=1,\ldots ,J$. Next, for each of the 100 parameter sets we simulated 1000 samples of *N* subjects responding to the *J* items and applied the CARP test procedure to this sample with nominal significance level $\alpha =.05$. Figure 2 shows modified boxplots of the rejection rates. As expected, the power increased with *N*, but variation was large due to the parameter sets. For $N=5000$, most parameter sets would have had power greater than .80.

**Table 4:** Rejection rates of CARP and CRS tests in three-dimensional cases with $N=$ 5,000.

*J*	$J_{1}$	$J_{2}$	$J_{3}$	CARP	CRS
12	2	2	8	**.154**	.064
12	3	3	6	**.373**	.148
12	4	4	4	**.541**	.398
12	5	5	2	.684	**.767**
24	2	2	20	**.130**	.061
24	3	3	18	**.285**	.060
24	4	4	16	**.476**	.092
24	5	5	14	**.622**	.171
24	6	6	12	**.732**	.240
24	7	7	10	**.801**	.493
24	8	8	8	**.855**	.729
24	9	9	6	.899	**.915**
24	10	10	4	.914	**.982**
24	11	11	2	.945	**.995**

Values in bold are the largest power in the row

Each row is based on 1000 samples.

## Additional Results for the CARP Tests

### Aggregation of CARP Tests across Item Pairs

We discuss how one can combine tests of multiple item pairs without prior selection. If one applies a CARP test to all item pairs of a scale, this produces a sequence of $J(J-1)/2$ *p*-values. To keep the family-wise Type I error rate (FWER) under control, a multiple testing correction may be in order. Alternatively, one could choose to control the false discovery rate (FDR), which generally leads to tests with higher power (Benjamini & Hochberg, 1995; Benjamini & Yekutieli, 2001). However, applying either correction method to all $J(J-1)/2$ *p*-values results in unnecessary loss of power if many of the involved covariances are positive. Instead, one may consider only the item pairs that have a negative conditional covariance in the training sample and test their conditional covariances in the test sample. We collect the set of item pairs $\left( i,j \right) ,i>j$ with a negative conditional covariance in the training sample in set $\mathcal {S}$, and let $S:=\left| \mathcal {S} \right|$ denote its size. Since this statistic is independent of the data in the test sample, one may apply the Bonferroni correction with this number; that is, reject the null hypothesis for pair (*i*, *j*) iff $(i,j)\in \mathcal {S}$ and $p_{ij}\le \alpha /S$. To check that this correction with *S* controls the FWER, assume that under the null hypothesis the distribution of each $p_{ij}$ dominates the *Uniform*(0, 1) distribution in the sense that $\mathbb {P}\left( p_{ij}\le x \right) \le x$ for all $x\in \mathbb {R}$ where $\mathbb {P}$ denotes the probability measure of the $p_{ij}$. This condition is called supra-uniformity by Ellis et al. (2020). The condition is satisfied if $p_{ij}$ dominates the *Uniform*(0, 1) distribution in likelihood ratio order (Whitt, 1980), and it is equivalent to $p_{ij}$ having a density that is increasing on the interval (0, 1). This is true if the test is conducted with $p_{ij}={\Phi }^{-1}\left( Z_{ij} \right)$ with $Z_{ij}\sim \mathcal {N}(\mu _{ij},1)$ and $\mu _{ij}\ge 0$. Let $\mathcal {R}_{ij}$ denote the event that the null hypothesis is rejected for pair (*i*, *j*), then, for a fixed pair (*i*, *j*) we have $\mathbb {P}\left( \mathcal {R}_{ij} \vert {(i,j)\in \mathcal {S}}\right) \le \alpha /S$ and $\mathbb {P}\left( \mathcal {R}_{ij} \vert {{\, \, \, }(i,j)\notin \mathcal {S}}\right) =0$; therefore, the FWER is

$$\begin{aligned} \mathbb {P}\left( {\bigcup _{i,j} {{\mathcal {R}}_{ij}}} \right) = \mathbb {E}\left( \mathbb {P} {\left( {\bigcup _{i,j} {{\mathcal {R}}_{ij}}|{\mathcal {S}}} \right) } \right) \le \mathbb {E}\left( {\Sigma _{i,j} \mathbb {P}\left( {{{\mathcal {R}}_{ij}} |{\mathcal {S}}} \right) } \right) \le \mathbb {E}\left( {S{\alpha \over S}} \right) = \alpha . \end{aligned}$$

(In $\mathbb {P}\left( {\bigcup _{i,j} {{\mathcal {R}}_{ij}}}|\mathcal {S} \right)$ and $\mathbb {P}\left( {{\mathcal {R}}_{ij}}|\mathcal {S}\right)$, $\mathcal {S}$ is treated as a random variable with outcomes that enumerate the power set of ${\{1,\ldots ,J\}}^{2}$.)

Alternatively, one could try to compound the *Z*-statistics in the formula

$$\begin{aligned} Z_{total}=\frac{\mathop {\sum }\nolimits _{(i,j)\in {\, }\mathcal {T}} Z_{ij} }{\sqrt{v} }, \end{aligned}$$

where $\mathcal {T}\subseteq \mathcal {S}$ is some further restricted subset of item pairs, and *v* is an estimate of the variance of the numerator. The $Z_{ij}$s are correlated, and to obtain *v* one should somehow estimate their average correlation; see Efron (2010), who discusses methods for this purpose. An advantage is that compounding can increase the power, as was concluded by Straat et al. (2016) for the CA tests of Rosenbaum (1984). We encourage future researchers to develop improved compounding rules.

### Comparison with Other Methods

So far, we compared CARP mathematically with other incomplete tests of CA, such as testing MTP$_{2}$, and we compared it in simulations with Rosenbaum’s (1984) CRS test, which also assesses CA. Next, we briefly touch upon the topics of parametric goodness-of-fit tests and discuss the difference of the CARP method and existing approaches to dimensionality assessment in nonparametric IRT. We notice that the CARP test has been developed for a single focal pair, so that aggregation over multiple item pairs is a topic for future research. The alternatives that we discuss typically aggregate over all item pairs. This is true for Chi-square and RMSEA in factor analysis, the statistics $Q_{2}$ (Van den Wollenberg, 1982), $R_{2}$ (Glas, 1988), and $M_{2}$ (Maydeu-Olivares & Joe, 2006) in logistic IRT models, and DETECT (Zhang & Stout, 1999b) in nonparametric IRT. Our discussion can therefore not include a quantitative comparison with respect to statistical power.

#### Comparison with Parametric Goodness-of-Fit Tests

Two reviewers suggested to compare the CARP procedure with a method where a parametric multidimensional model is fitted first and then a parametric unidimensional model is compared with it, using a goodness-of-fit test such as a likelihood-ratio test. We will call this method the *parametric goodness-of-fit comparison* (PGC). Examples of this are (1) testing multiple linear factor models and compare their chi-square statistics, assuming normal distributions, or compare their Chi-squares, RMSEAs or eigenvalues. We mention this possibility because of its popularity in psychology; (2) similar but using logistic IRT models instead of linear factor models (e.g., Bartolucci, 2007; Christensen et al. 2002; 3) testing different latent class models (e.g., Bartolucci et al. 2017; Ligtvoet & Vermunt 2012; Van Onna, 2002; Vermunt, 2001). The idea is that a latent class model can approximate nonparametric multidimensional and unidimensional models if the number of latent classes is large enough; and (4) testing monotonic polynomial models (Falk & Cai, 2016). These models can be used to approximate multidimensional and unidimensional models if the number of polynomial terms is large enough, and therefore a similar strategy can be used.

We find this approach interesting, but we are not yet convinced that in the long run it is more helpful than our approach, which is focussed on critical data patterns such as negative covariances rather than comparing goodness-of-fit statistics. Our restraints concerning the PGC methods are the following. First, it is generally difficult to know how many dimensions the multidimensional model should have, and how this influences the decision on the unidimensional model. Second, it is unclear to which extent the auxiliary assumptions (linearity, logistic response function, normality, number of latent classes, number of polynomial terms) influence the goodness-of-fit of the unidimensional model. Third, if a goodness-of-fit test indicates that the unidimensional model is wrong, it might not be clear which items are causing the problem. For some models, item-fit statistics have been proposed (e.g., Sijtsma & Van der Ark, 2021) that must be used in combination with statistics assessing the fit of sets of items. Another variant is that an alternative unidimensional model must be chosen, but then the large array of possibilities provides a new choice problem (which model is the most obvious choice?) and corresponding analysis problem (how to avoid endless trial and error?). We conclude that the application of methodologies other than the one we study in this article comes with complexities hindering their straightforward use as well as a simple comparison with our CARP methodology.

#### Comparison with Item Selection Procedures in Nonparametric IRT

In the context of nonparametric IRT, several procedures have been proposed to assess the dimensionality of an item set. The automated item selection procedure (AISP; Mokken, 1971; Sijtsma & Molenaar, 2002) uses a bottom-up algorithm to select items in unidimensional subsets based on a definition of a scale that uses non-negative inter-item covariances and positive scalability coefficients. Straat et al. (2013) proposed a genetic algorithm to replace and remedy some of the peculiarities of the AISP. The goal of both procedures is to have as many items possible in the first scale, as many from the remaining items—if available—in the second scale, and so on. Zhang & Stout (1999b, p. 239) defined the “bias-corrected estimator for the theoretical DETECT index” as a weighted average of covariances of the form $Cov\left( {X_{i},X_{j}} \vert T\right)$, with sum score $T=\sum \nolimits _i X_{i}$, and $Cov\left( {X_{i},X_{j}} \vert R_{ij}\right)$, with rest score $R_{ij}=\sum \nolimits _{k\ne i,j} X_{k}$, where the DETECT weights are such that the pair (*i*, *j*) contributes if and only if both items are in the same cluster. Next, Zhang and Stout try to find the partition that maximizes this index using a heuristic procedure. Roussos, Stout, and Marden (1998) proposed an agglomerative hierarchical cluster analysis for finding subsets of items, using the software package HCA/CCPROX. The procedure provided the choice between different statistics, including covariances conditional on rest scores not including items known to be in already formed clusters, for assessing the relationship between items, and different agglomerative hierarchical clustering methods. They did not use a formal criterion for identifying a final solution but rather left this to the researcher to decide, for example, based on theoretical expectations of the item set’s dimensionality. The DIMTEST procedure assesses the hypothesized unidimensionality of a user-specified item set (Nandakumar & Stout, 1993; Stout, 1987). Thus, unlike the other procedures, DIMTEST is confirmatory and cannot directly be used to partition items in different clusters in an exploratory analysis. Several variations on the original procedure have been proposed; see Stout et al. (2001) and Kieftenbeld & Nandakumar (2015). Van Abswoude, Van der Ark, and Sijtsma (2004) systematically compared the methods.

The CARP procedure is different from these and other item selection procedures proposed in the nonparametric IRT context (e.g., Brusco, Köhn, & Steinley, 2015). It shares with several of these procedures a certain open-endedness caused by the complexities typical of a fine-grained analysis of the data involving many item pairs or item subsets and subdivisions of the sample into score groups, dealing with finite sample sizes and empty or near-empty cells in contingency tables, and combining many detailed results into one useful conclusion about the dimensionality of an item set. Because so many arbitrary researcher decisions are needed to obtain a result, not only for the CARP procedure but also for other procedures many precautions are needed to be able to compare them thoroughly. This is a project requiring a separate study.

## Discussion

We developed the CARP test, which often distinguishes data generated by a two-dimensional model from data generated by a unidimensional monotone model, even if the data are MTP$_{2}$ and have MM. The test uses CA and can be viewed as a generalization of Rosenbaum’s (1984) proposal to test the covariance of each item pair conditionally on their unweighted rest score (the CRS test). The CARP test conditions on a weighted rest score, where the weights are based on regression analyses in a training sample consisting of $20\%$ to $50\%$ of the total sample. Each of the items in a focal pair (*i*, *j*) is used as dependent variable in a linear regression analysis that predicts them from the remaining items. The sum of the two predicted scores is computed in the test sample and is used as the weighted rest score. The weighted rest score divides the test sample into deciles and a directional Mantel–Haenszel test tests whether the covariance of (*i*, *j*) is nonnegative in each decile group.

Data generated by means of unidimensional logistic models showed that the Type I error rate is under control, even if the overall inter-item correlations are 0. Simulations with two-dimensional logistic models showed the power of the CARP test exceeds the power of the CRS test if one dimension has three times more items than the other dimension. Simulations with three-dimensional logistic models showed the power of the CARP test exceeds the power of the CRS test if the third dimension has at least a third of the items. In the extreme two-dimensional case, where both dimensions have the same number of items with equal loadings and difficulty parameters for all items, the CARP test converges to the CRS test as the sample size increases. Thus, in comparison with Rosenbaum’s (1984) CRS test, our CARP test gains power in a variety of multidimensional cases at the cost of losing some power in extreme two-dimensional cases with equally important dimensions. Because tests are usually aimed at being unidimensional, most of the items indeed resulting in targeting this dimension, the results for the CARP test are positive.

We explored for multiple focal items that compounding their test statistics can increase the power. The CARP method looks promising, but as with any newly developed method it also raises questions for future research. First, what are the optimal values of $\ell$ (the size of the training sample) and *m* (the number of groups in conditioning), and how do these values depend on *N* and *J*? Second, in the cross-validation, rather than drawing one training sample one might repeat drawing and then aggregate the results over draws, thus reducing the variability of the outcomes. Which aggregation rules are suitable? Third, how can one compound test results for multiple item pairs? Fourth, a more elaborate study of the dependence of the power on the number of items, the number of dimensions, the shape of the response function (logistic or other), and the item parameters could be done. Fifth, the CARP inequalities also hold for polytomous items. Which test procedures are most useful? Rosenbaum (1984, p. 429) provides suggestions. Sixth, how does the power profile of the CARP test compare to the semiparametric methods of Bartolucci (2007) and Falk and Cai (2016)?.

In our analysis, we assumed a priori that conditional independence holds, which is consistent with the fact that for a finite number of binary items, without other restrictions, conditional independence is a “vacuous assumption” (Holland & Rosenbaum, 1986, p. 1525). Moreover, assuming monotonicity, we developed the CARP test as a test of unidimensionality versus multidimensionality. However, if the CARP test points to a violation of MH, this cannot be attributed to a single assumption. An alternative model may thus assume local dependence or correlated errors instead of multidimensionality.

The CARP method can be a useful addition to the existing methods for testing MH and detecting multidimensionality in monotone models. It may help answer a fundamental empirical question without relying on features of parametric models that are irrelevant to the research question. We already mentioned the present CHC intelligence representation using multiple factors (Wasserman, 2019) that is based on parametric—mostly linear—models. This choice is mathematically convenient but may be irrelevant for distinguishing the factors and damaging when it dominates the data analysis. A significant negative covariance obtained in a CARP test would demonstrate that the distinction between intelligence factors is not an artifact of the parametric assumptions, and it would rule out every unidimensional monotone model for intelligence. This is another topic for future research.

### Supplementary Information

Below is the link to the electronic supplementary material.

Supplementary file 1 (xlsx 78 KB)

Supplementary file 2 (txt 2 KB)

## Appendix A

In this appendix, we state the precise definitions of the various models and conditions that are relevant here. Consider a vector of binary manifest variables, ${{\textbf {X}}}=\left( X_{1}, \ldots ,X_{J} \right)$. Variable $X_{i}$ represents the scores (1 $=$ correct, 0 $=$ incorrect) subjects obtained on the *i*-th item. Suppose that in the probability space of ${{\textbf {X}}}$ there is some random vector, ${\varvec{\Theta }=}\left( {\Theta }_{1}, \ldots ,{\Theta }_{D} \right)$, which represents the latent variables. We will use the following conditions (Holland & Rosenbaum, 1986; Mokken, 1971; Rosenbaum, 1984), adapted to binary manifest variables:

1. (conditional independence). ${\textbf {X}}$ is *conditionally independent* given ${\varvec{\Theta }}$ if $P({{\textbf {X}}}={{\textbf {x}}}\vert {\, \varvec{\Theta })=}\prod _{i=1}^J {P(X_{i}=x_{i}\vert {\, \varvec{\Theta })}}$ for all ${{\textbf {x}}}\in {\{0,1\}}^{J}$.
2. (monotonicity). ${{\textbf {X}}}$ is *monotone* with ${\varvec{\Theta }}$ if $P(X_{i}=1\vert {\, \varvec{\Theta })}$ is monotonically increasing in each coordinate of ${\varvec{\Theta }}$ for all $i=1,\ldots ,J$.
3. (unidimensionality). ${\varvec{\Theta }}$ is *unidimensional* if $D=1$.

### Definition 1

*(monotone latent variable model;* Holland & Rosenbaum, 1986). $({{\textbf {X}}}\mathbf {,\, }{\varvec{\Theta }})$ is a *monotone latent variable *(MLV) model if ${{\textbf {X}}}$ is conditionally independent given ${\varvec{\Theta }}$ and ${{\textbf {X}}}$ is monotone with ${\varvec{\Theta }}$. If, additionally, ${\varvec{\Theta }}$ is unidimensional, then $({{\textbf {X}}}\mathbf {,\, }{\varvec{\Theta }})$ is a unidimensional monotone latent variable model.

Mokken (1971), Mokken and Lewis (1982) introduced the unidimensional monotone latent variable model for binary items as the monotone homogeneity (MH) model. Ellis and Junker (1997) reformulated Definition 1 such that it is a property of ${{\textbf {X}}}$ rather than $({{\textbf {X}}}\mathbf {,\, }{\varvec{\Theta }})$:

### Definition 2

*(monotone homogeneity)*.  $\varvec{X}$ satisfies a *unidimensional monotone latent variable model* or *monotone homogeneity *(MH) if there exists a unidimensional variable ${\Theta }$ such that ${{\textbf {X}}}$ is conditionally independent given ${\Theta }$ and ${{\textbf {X}}}$ is monotone with ${\Theta }$.

Ellis (2015) studied a narrower formulation of monotone models that will be called ‘monotone factor models’ here. His assumptions can be rephrased as follows:

1. $X_{i}=\phi _{i}\left( \eta _{i}+\upvarepsilon _{i} \right)$ for $i=1,\ldots ,J$, where each $\phi _{i}$ is an increasing function (henceforth, called a response function), and $\eta _{i}$ and $\upvarepsilon _{i}$ are latent variables. We write$\, {{\varvec{\eta }}=(\eta }_{1},\ldots ,\eta _{J})$.
2. ${\varvec{\eta }}=\psi ({\varvec{\lambda \Theta }}\mathbf {)}$, where ${\varvec{\Theta }}$ is a multivariate vector with independent components, ${\varvec{\lambda }}$ is a real matrix, and $\psi$ is an increasing function.
3. The latent variables $\upvarepsilon _{1},\upvarepsilon _{2},\ldots$ are independent from each other and independent of ${\varvec{\Theta }}$.
4. The $\upvarepsilon _{i}$ have densities that are Pólya frequency functions of order 2 (PF2; Efron, 1965).
5. ${\varvec{\lambda }}$ has a simple structure where every manifest variable loads positive on one factor and zero on the other factors.

Assumption MF1 means that the item responses are a dichotomization of underlying item-specific latent variables $\eta _{i}+\upvarepsilon _{i}$ that one may view as latent responses. This has been discussed earlier in the context of the normal ogive model (Takane & de Leeuw, 1987), but normality is not assumed here. Assumption MF2 specifies that the common parts $\eta _{i}$ of the latent responses have a monotone relationship with the same underlying, more fundamental factors in ${\varvec{\Theta }}$. These underlying factors should be independent. MF3 states that the unique factors or error variables $\upvarepsilon _{i}$ are independent, which is comparable to conditional independence. Assumption MF4 is equivalent to the assumption that the distributions of $\upvarepsilon _{i}$ are log-concave (e.g., Saumard & Wellner, proposition 2.3) or strongly unimodal (Walther, 2009, p. 320). This assumption is satisfied in many models, as this includes normal, uniform, gamma, beta, and logistic densities. Assumption MF5 requires a simple structure of the factor loadings.

### Definition 3

*(monotone factor model)*. ${{\textbf {X}}}$ satisfies a *monotone factor model* (MFM) if there are ${\varvec{\lambda }}$, ${\varvec{\Theta }}$, and $\upvarepsilon _{1},\upvarepsilon _{2},\ldots$ such that MF1 – MF5 hold.

In other words, an MFM has factors that are independent, nonnegative loadings with a simple structure, strongly unimodal latent errors, and increasing response functions.

We will now define the concept of *multivariate positivity of order 2 *(MTP$$_{2})$$ and related concepts. Let $$\chi :=\times _{i=1}^{m}\chi _{m}$$ be a product lattice in $$R^{m}$$. Let the lattice operators be defined as, for all $${{\textbf {x}},{\textbf {y}}{\in }}\chi $$:

$$\begin{aligned}{} & {} {{\textbf {x}}}\vee {{\textbf {y}}:=}\left( \textrm{max}\left\{ x_{1},y_{1} \right\} ,\ldots ,\textrm{max}\left\{ x_{m},y_{m} \right\} \right) \\{} & {} {{{\textbf {x}}\,{\wedge }\,{\textbf {y}}}:=}\left( \textrm{min}\left\{ x_{1},y_{1} \right\} ,\ldots ,\textrm{min}\left\{ x_{m},y_{m} \right\} \right) \end{aligned}$$

### Definition 4

(MTP$_{\textbf{2}}$; Karlin and Rinott (1980)*)*. A random vector ${{\textbf {X}}}$ on product lattice $\chi$ is *MTP*$_{2}$ if it has density *f*, and for all ${{\textbf {x}},{\textbf {y}}{\in }}\chi$,

$$\begin{aligned} \begin{aligned} f\left( {{{\textbf {x}}}}\vee {{{\textbf {y}}}}\right) f\left( {{{\textbf {x}}}{\wedge }{} {{\textbf {y}}}}\right) \ge f\left( {{{\textbf {x}}}}\right) f\left( {{{\textbf {y}}}}\right) \end{aligned} \end{aligned}$$

MTP$_{2}$ generalizes the idea of a positive correlation and is also known as supermodularity, the FKG condition (Denuit et al. 2005) or affiliation (Milgrom & Weber, 1982). Denote the correlation between variables *X* and *Y* by $\rho _{XY}$.

### Definition 5

*(**nonnegative partial correlations, NPC;* Ellis (2014)*)*. ${{\textbf {X}}}$ has *nonnegative partial correlations* (NPC) if for every triplet (*X*, *Y*, *Z*) of variables in ${{\textbf {X}}}$, $\rho _{XY}\ge \rho _{XZ}\rho _{ZY}$.

### Definition 6

*(**nonnegative covariances, NNC;* Mokken (1971)*)*. ${\textbf {X}}$ has *nonnegative covariance* (NNC) if $\textrm{Cov}(X_{i},X_{j})\ge 0$ for every pair $(X_{i},X_{j})$ of variables in ${{\textbf {X}}}$.

For any item *i*, we define the rest score as $X_{-i}=\left( \mathop {\sum }\nolimits _{j=1}^J X_{j} \right) -X_{i}$; that is, the sum score with the score of item *i* omitted.

### Definition 7

*(**manifest monotonicity, MM;* Junker (1993)*)*. ${{\textbf {X}}}$ has *manifest monotonicity* (MM) if $\mathbb {E}(X_{i}\vert X_{-i})$ is increasing in $X_{-i}$ for each $i=1,\ldots ,J$.

The following proposition is mostly implied by Corollary 3 of Ellis (2015), except that we do not require ${\varvec{\Theta }}$ to be PF2 here.

### Proposition A1

If ${\textbf {X}}$ satisfies a MFM, then ${{\textbf {X}}}$ is MTP$_{2}$.

### Proof

This can be derived from three elementary facts about MTP$_{2}$ and PF2 variables: (1) independent variables are MTP$_{2}$ (Karlin & Rinott, 1980, proposition 3.5), (2) MTP$_{2}$ is preserved by increasing transformations (Karlin & Rinott, 1980, proposition 3.6; Ellis, 2015, proposition 8), and (3) the sum of MTP$_{2}$ and independent PF2 variables is MTP$_{2}$ (Karlin & Rinott, 1980, proposition 3.7). Consequently, assuming MF1-MF5, we obtain that ${\varvec{\Theta }}$ is MTP$_{2}$ because it has independent components, ${\varvec{\eta }}$ is MTP$_{2}$ because it is an increasing function of ${\varvec{\Theta }}$, and ${\varvec{\eta }}\mathbf {+}{\varvec{\upvarepsilon }}$ is MTP$_{2}$ because it is a sum of MTP$_{2}$ and independent PF2 variables. ${\textbf {X}}$ is MTP$_{2}$ because it is an increasing transformation of ${\varvec{\eta } +\varvec{\upvarepsilon }}$. $\square$

## Appendix B

In this appendix, we will prove Theorem 1, which implies that MFMs satisfy MM. For ease of notation, we will first extend the definition of conditional expectations such as $\mathbb {E}\left( X_{i}\vert \sum \nolimits _{j=1,j\ne i}^J X_{j} =r \right)$. Suppose *R* is a bounded nonnegative integer valued variable and *X* is a binary variable. If $P\left( R=r \right) =0$, then $\mathbb {E}\left( X\vert R=r \right)$ is not uniquely defined, and we extend the definition of $\mathbb {E}\left( X\vert R=r \right)$ to all $r\in \mathbb {Z}$, including cases with $P\left( R=r \right) =0$, such that it remains increasing in $r\in \mathbb {Z}$, provided that it is increasing to begin with. The precise values are not relevant, and one possibility is defining

$$\mathbb {E}\left( X\vert R=r \right) :=0$$ if $$P\left( R\le r \right) =0$$,

$$\mathbb {E}\left( X\vert R=r \right) :=1$$ if $$P\left( R\ge r \right) =0$$, and

$$\mathbb {E}\left( X\vert R=r \right) :=\frac{\mathbb {E}\left( X\vert R=r^{-} \right) +\mathbb {E}\left( X\vert R=r^{+} \right) }{2}$$ if $$P\left( R=r \right) =0$$, $$P\left( R\le r \right) >0$$, and $$P\left( R\ge r \right) >0$$

where $r^{-}=\sup \{s \in \mathbb {Z}| s < r, P\left( R=s \right) >0\}$ and $r^{+}=\inf \{s \in \mathbb {Z}| s> r, P\left( R=s \right) >0\}$. In other words, if $\mathbb {E}\left( X\vert R=r \right)$ is undefined for some value of *r*, then we set it equal to 0 if $r<\textrm{min}(R)$, equal to 1 if $r>\textrm{max}(R)$, and equal to the average of the two surrounding values otherwise. Similarly, if *S* is another random variable, we can define $\mathbb {E}\left( X\vert R=r,S \right) :=\mathbb {E}\left( X\vert R=r \right)$ in cases with $P\left( R=r \right) =0$, and $\mathbb {E}\left( X\vert R+S=t,S=s \right)$ can be defined accordingly.

### Lemma 1

Let *R* and *S* be bounded nonnegative integer valued variables and let *X* be a variable with finite expectation. If $\mathbb {E}\left( X\vert R=r \right)$ is increasing in $r=0,\, 1,\ldots$ and (*X*, *R*) is independent of *S*, then $\mathbb {E}\left( X\vert R+S=t \right)$ is increasing in $t=0,\, 1,\ldots$.

### Proof

Since *S* is independent of *X* and *R*, we have for every $s=0,\, 1,\ldots ,\textrm{max}(S)$,

$$\begin{aligned}{} & {} \mathbb {E}\left( X\vert R+S=t+1,S=s \right) =\mathbb {E}\left( X\vert R=t+1-s,S=s \right) =\mathbb {E}\left( X\vert R=t+1-s \right) \ge \\{} & {} \quad \mathbb {E}\left( X\vert R=t-s \right) =\mathbb {E}\left( X\vert R=t-s,S=s \right) =\mathbb {E}\left( X\vert R+S=t,S=s \right) \end{aligned}$$

Therefore,

$$\begin{aligned}{} & {} \mathbb {E}\left( X\vert R+S=t+1 \right) =\mathop {\sum }\limits _{s=0}^{\textrm{max}(S)} {E\left( X\vert R+S=t+1,S=s \right) } P\left( S=s \right) \ge \\{} & {} \mathop {\sum }\nolimits _{s=0}^{\textrm{max}(S)} {E\left( X\vert R+S=t,S=s \right) P\left( S=s \right) } =\mathbb {E}\left( X\vert R+S=t \right) \end{aligned}$$

$$\square $$

### Theorem 1

Suppose that the set of test items $\left\{ X_{1}, \ldots ,X_{J} \right\} \,$can be divided into disjoint subtests such that different subtests are independent while items within the same subtest satisfy MH. Then, MM holds for the entire set of test items, that is, $\mathbb {E}\left( X_{i}\vert \sum \nolimits _{j=1,j\ne i}^J X_{j} =t \right)$ is increasing in   *t* for each $i=1,\ldots ,J$*.*

### Proof

Consider an arbitrary item for which MM should be established. Without loss of generality, we may assume that this item is $X_{1}$ and that the first subtest is $\left( X_{1}, \ldots ,X_{k} \right)$. With $R:=\sum \nolimits _{i=2}^k X_{i}$ and $S:=\sum \nolimits _{i=k+1}^J X_{i}$ we can write $\mathbb {E}\left( X_{1}\vert \sum \nolimits _{i=2}^J X_{i} =t \right) =\mathbb {E}\left( X_{1}\vert R+S=t \right)$, where $X_{1}$ and *R* are independent of *S*. Since $\left( X_{1}, \ldots ,X_{k} \right)$ satisfies MH, it must also satisfy MM, that is, $\mathbb {E}\left( X_{1}\vert R=r \right)$ is increasing in *r*. Using Lemma 1, we obtain that $\mathbb {E}\left( X_{1}\vert R+S=t \right)$ is increasing in *t*. $\square$

### Theorem 2

Suppose that the set of test items $\left\{ X_{1}, \ldots ,X_{J} \right\} \,$can be divided into two disjoint subtests such that different subtests are independent while items within the same subtest satisfy MH. If two items $X_{i}$ and $X_{j}$ belong to different subtests, then $\mathbb {E}\left( Cov\left( {X_{i},{\, }X_{j}} \vert \mathop {\sum }\nolimits _{k\ne i,j}^J X_{k} \right) \right) \le 0.$

### Proof

Without loss of generality, we can assume, for notational convenience, that the first subtest is $\left\{ X_{1}, \ldots ,X_{J_{1}} \right\}$ and the second subtest is $\left\{ X_{J_{1}+1}, \ldots ,X_{J} \right\}$, and that $i=1$ and $j=J$. As a consequence of MM, $\mathbb {E}\left( X_{1}\vert \mathop {\sum }\nolimits _{k=2}^{J_{1}} X_{k} =t \right)$ is increasing in *t*. Now, apply Lemma 1 with $X=X_{1},\, R=\mathop {\sum }\nolimits _{k=2}^{J_{1}} X_{k}$ and $S=\mathop {\sum }\nolimits _{k=J_{1}+1}^{J-1} X_{k}$. It follows that $\mathbb {E}\left( X_{1}\vert \mathop {\sum }\nolimits _{k=2}^{J-1} X_{k} =t \right)$ is increasing in *t*. Similarly, $\mathbb {E}\left( X_{J}\vert \mathop {\sum }\nolimits _{k=2}^{J-1} X_{k} =t \right)$ is increasing in *t* too. Therefore, $Cov\left( \mathbb {E}\left( X_{1}\vert \mathop {\sum }\nolimits _{k=2}^{J-1} X_{k} \right) ,\mathbb {E}\left( X_{J}\vert \mathop {\sum }\nolimits _{k=2}^{J-1} X_{k} \right) \right) \ge 0$. Since $Cov\left( X_{1},X_{J} \right) =0$ and, by the law of total covariance,

$$\begin{aligned} Cov\left( X_{1},X_{J} \right)= & {} \mathbb {E}\left( Cov\left( {X_{1},{\, }X_{J}} \vert \mathop {\sum }\nolimits _{k=2}^{J-1} X_{k} \right) \right) \\+ & {} Cov\left( \mathbb {E}\left( X_{1} \vert \mathop {\sum }\nolimits _{k=2}^{J-1} X_{k} \right) ,\mathbb {E}\left( X_{J} \vert \mathop {\sum }\nolimits _{k=2}^{J-1} X_{k} \right) \right) , \end{aligned}$$

it follows that $\mathbb {E}\left( Cov\left( {X_{1},{\, }X_{J}} \vert \mathop {\sum }\nolimits _{k=2}^{J-1} X_{k} \right) \right) \le 0$. $\square$

This can be generalized to weighted sum scores, provided that MM still holds with respect to the weighted sum scores of the subtests, as stated in the following lemma and theorem.

### Lemma 2

Let *R* and *S* be real valued random variables with finite range and let *X* be a variable with finite expectation. If $\mathbb {E}\left( X\vert R=r \right)$ is increasing in $r,\, \forall r\in \mathbb {R},$ and (*X*, *R*) is independent of *S*, then $\mathbb {E}\left( X\vert R+S=t \right)$ is increasing in $t, \forall t\in \mathbb {R}$.

### Proof

Extend the definition of $\mathbb {E}\left( X\vert R=r \right)$ and $\mathbb {E}\left( X\vert R=r,S \right)$ to all $r\in \mathbb {R}$, just as we did prior to Lemma 1. Since *S* is independent of *X* and *R*, we have for every $s\in \mathbb {R}$, $\delta >0$:

$$\begin{aligned}{} & {} \mathbb {E}\left( X\vert R+S=t+\delta ,S=s \right) =\mathbb {E}\left( X\vert R=t+\delta -s,S=s \right) =\mathbb {E}\left( X\vert R=t+\delta -s \right) \ge \\{} & {} \quad \mathbb {E}\left( X\vert R=t-s \right) =\mathbb {E}\left( X\vert R=t-s,S=s \right) =\mathbb {E}\left( X\vert R+S=t,S=s \right) \end{aligned}$$

Therefore,

$$\begin{aligned} \mathbb {E}\left( X\vert R+S=t+\delta \right) =\mathop {\sum }\limits _{s\, \in \, range(S)} {\mathbb {E}\left( X\vert R+S=t+\delta ,S=s \right) } P\left( S=s \right) \ge \\ \mathop {\sum }\nolimits _{s\, \in \, range(S)} {\mathbb {E}\left( X\vert R+S=t,S=s \right) P\left( S=s \right) } =\mathbb {E}\left( X\vert R+S=t \right) \end{aligned}$$

$$\square $$

### Theorem 3

Suppose that the set of test items $\left\{ X_{1}, \ldots ,X_{J} \right\} \,$can be divided into two disjoint subtests $\left\{ X_{1}, \ldots ,X_{J_{1}} \right\}$ and $\left\{ X_{J_{1}+1}, \ldots ,X_{J} \right\}$, such that different subtests are independent. Let $a_{1},\ldots ,a_{J}\in \mathbb {R}$ be such that both $\mathbb {E}\left( X_{i} \vert {\mathop {\sum }\nolimits _{k=2}^{J_{1}} {a_{k}X_{k}} =t}\right)$ and $\mathbb {E}\left( X_{j} \vert {\mathop {\sum }\nolimits _{k=J_{1}+1}^{J-1} {a_{k}X_{k}} =t}\right)$ are increasing in *t*. Then, $\mathbb {E}\left( Cov\left( {X_{1},{\, }X_{J}} \vert \mathop {\sum }\nolimits _{k=2}^{J-1} {a_{k}X}_{k} \right) \right) \, \le 0$.

### Proof

By Lemma 2, $\left( X_{1} \vert {\mathop {\sum }\nolimits _{k=2}^{J-1} {a_{k}X_{k}} =t}\right)$ and $\mathbb {E}\left( X_{J} \vert {\mathop {\sum }\nolimits _{k=2}^{J-1} {a_{k}X_{k}} =t}\right)$ are both increasing in *t*. Therefore, their covariance is nonnegative, i.e., C$ov\left( \mathbb {E}\left( X_{1}\vert \mathop {\sum }\nolimits _{k=2}^{J-1} {a_{k}X}_{k} \right) ,\mathbb {E}\left( X_{J}\vert \mathop {\sum }\nolimits _{k=2}^{J-1} {a_{k}X_{k}} \right) \right) \ge 0$. The hypothesis of the theorem implies that $Cov\left( X_{1},X_{J} \right) =0$, and the conclusion follows by the law of total covariance. $\square$
